# Glucagon-like peptide (GLP) -2 improved colonizing bacteria and reduced severity of ulcerative colitis by enhancing the diversity and abundance of intestinal mucosa

**DOI:** 10.1080/21655979.2021.1958600

**Published:** 2021-08-17

**Authors:** Dongyue Li, Youlin Yang, Xunhai Yin, Yang Liu, Hongyu Xu, Yu Ni, Ping Hang, Sijia Niu, Huichao Zhang, Wenbo Ding, Hongyu Kuang

**Affiliations:** a Department of Gastroenterology, The First Affiliated Hospital, Harbin Medical University, Harbin Heilong Jiang Province, People’s Republic of China; b Department of Endocrinology, The First Affiliated Hospital, Harbin Medical University, Harbin Heilong Jiang Province, People’s Republic of China

**Keywords:** Glucagon-like peptide 2, high-throughput 16S RNA sequencing, glucose metabolism analysis, intestinal microbiota, ulcerative colitis

## Abstract

The global incidence of ulcerative colitis (UC) continues to increase while it’s clinical cure rate remains low. Intestinal mucosal ulcers have segmental distribution and variable severity. Intestinal bacteria are closely related to intestinal immunity and metabolism; however, the relationship between intestinal microbiome profile and the occurrence of UC, as well as the contribution of glucose metabolism, are not well understood. This was investigated in the present study using mucosal biopsies from patients with UC and healthy control subjects. We performed high throughput 16S rRNA gene sequencing to estimate microbiota composition and abundance as well as their association with clinical indices such as lesion severity. The results showed that the diversity and abundance of intestinal microbiota were significantly lower in patients with UC than in healthy subjects; however, these were unrelated to ulcer severity. Serum glucagon-like peptide 2 (GLP-2) level was associated with reduced microbiota diversity and abundance in UC. These results indicate that colonization by specific microbiota is not the main determinant of pathologic status in UC. Additionally, therapeutic strategies that increase GLP-2 levels in intestinal mucosa may be effective in the treatment of UC.

## Graphical abstract

**Figure:**
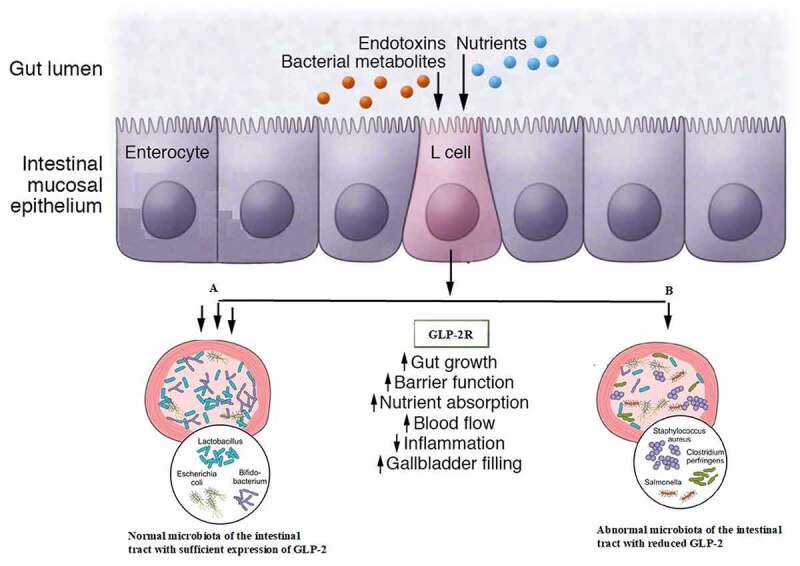

## Introduction

Ulcerative colitis (UC) is a disease characterized by inflammation of the intestinal tract. The clinical cure rate of UC is low; however, treatments such as probiotic preparations and fecal microbiota transplantation have had some clinical success, highlighting the central role of gut microbiota in UC pathology [1–3]Abnormal immune responses and destruction of the mucosal-epithelial barrier in UC have been attributed to gut bacteria [4–6]Gut microbiome composition is altered in UC, with significant reductions observed in the abundance of *Bifidobacterium* and *Lactobacillus* [7,8]and increased numbers of pathogenic bacteria such as *Salmonella* spp., *Thyrobacterium* spp., and *Bacillus difficile* that result in the disruption of intestinal homeostasis. The contribution of gut microbiota to UC severity is acknowledged in UC treatment guidelines [9]

In clinical practice, we have observed that UC does not spread past the cecum. The distribution and progression of UC vary among individuals for reasons that are unclear. Inflammation in intestinal tissue is related to the host’s age as well as the location and composition of microbiota [10] and is correlated with the clinical features of UC.

Most studies on intestinal microbiota use fecal samples [11] which have certain advantages. Firstly, they reflect the overall status of the intestinal environment. Secondly, sample collection is simple and noninvasive. A disadvantage of fecal samples is that they are influenced by the living environment and dietary habits of an individual and are susceptible to environmental contaminants during collection. Moreover, they do not provide a detailed view of local conditions in the intestine that can affect lesions at specific sites. In contrast, mucosal biopsies can reveal local flora distribution in the intestinal mucosa.

UC is frequently accompanied by abnormal glucose metabolism, which has been attributed to hormonal imbalance or metabolic disorders. Dysregulation of glucose metabolism in UC has been linked to the intestinal microbiome profile [12,13]. Thus, changes in gut microbiota composition or abundance may affect inflammation and glucose metabolism in the intestine. Glucagon-like peptide 2 (GLP-2) is a peptide hormone that has a variety of beneficial effects on the intestine, such as inducing the expansion of mucosal surface area by stimulating crypt cell proliferation and promoting nutrient processing and absorption [14] GLP-2 was shown to exert a protective effect on intestinal mucosa [15,16]

The distribution and severity of lesions in UC is related to colonization of intestinal mucosa by specific microbiota and is influenced by the microenvironment, including processes such as glucose metabolism [17,18]. To test this hypothesis, in this study, we carried out microbiome profiling of mucosal lesions of varying degrees of severity obtained from patients with UC and intestinal mucosal tissue from healthy control subjects in order to determine whether changes in gut microbiota are associated with clinical manifestations and progression of UC.

## Materials and methods

### Study population

The study enrolled 11 patients with UC and segmental distribution of lesions who had not received probiotic or antibiotic treatment in the previous 4 weeks. All cases met the 2012 diagnostic criteria of the Chinese Medical Association’s Digestive Credit Inflammatory Bowel Disease Collaboration Group (Chinese Medical Association,2012) and were assigned a Mayo score. There were 3 severe and 4 mild cases of UC. According to UC treatment guidelines, the patients underwent endoscopic biopsy of the terminal ileum; severe and junctional lesions, as well as relatively normal areas of tissue, were collected along with a tissue sample from the rectum and a blood sample. Patients with severe heart disease or high blood pressure, pregnant women, and patients with contraindications for colonoscopy were excluded. The healthy control group comprised 9 subjects; in compliance with international ethics standards, the subjects underwent a biopsy for eosinophilic enteritis at the same hospital, and mucosal samples from their biopsies were confirmed to be normal by pathological examination (Table 1). Tissue samples were obtained from terminal ileum and rectum, and corresponding blood samples were also collected. In order to maintain consistency, all samples were collected by the same investigator based on the endoscopic diagnosis. There were no significant differences between UC patients and healthy control subjects in terms of age and BMI.

**Table 1.** Patients demographics and clinical characteristics

Number of (patients No.)	Gender	Age	Height	Weight	Medical history	Relative normal	Critical point	Diseased (Dissected) region*	Terminal ileum**	Extent of disease	Clinical type
3	female	47	162	55 kg	NO	3 C	3D	3E	3A	0–30	Mild
4	male	51	176	64 kg	NO	4B	4D	4E	4A	0–60	Severe
5	male	52	174	58 kg	NO	5 C	5B	5A	5D	0–45	Severe
12	female	65	162	54 kg	NO	12B	12 C	12D	12A	0–50	Mild
13	male	49	176	70 kg	NO	13B	13 C	13E	13A	0–45	Mild
14	male	34	178	68 kg	NO	14B	14 C	14E	14A	0–25	Severe
16	female	33	163	58 kg	NO	16B	16 C	16D	16A	0–70	Mild
Normal (control)								Rectum	Terminal ileum		
40	female	61	162	55 kg	NO	NA	NA	40B	40A	NA	NA
41	female	44	165	68 kg	NO	NA	NA	41B	41A	NA	NA
42	female	49	174	70 kg	NO	NA	NA	42B	42A	NA	NA
43	male	55	178	68 kg	NO	NA	NA	43B	43A	NA	NA
44	male	69	175	74 kg	NO	NA	NA	44B	44A	NA	NA

*Atlas of … .

**Atlas of … .

Lesion severity in UC patient samples was confirmed by histopathologic examination. Quality control of the samples was performed, and those that were of low quality were excluded from the analysis. 16S rRNA gene sequencing was performed for 12 of the 20 patients (36 different samples of intestinal mucosa). The study was approved by the Research Ethics Committee of the First Affiliated Hospital of Harbin Medical University and was carried out in accordance with the ethical principles outlined in the Declaration of Helsinki. Written and informed consent was obtained from each participant.

## Experimental procedures

### Serum ELISA, and immunohistochemistry

Serum GLP-2 level was determined by ELISA using a commercial kit (Jianglai Biological Technology Co, Shanghai, China, cat. no. JL19998) according to the manufacturer’s instructions. The hematoxylin and eosin (H&E) staining. and Immunohistochemical detection of GLP-2 in intestinal mucosa was performed according to standard procedures using an anti-GLP-2 antibody (Jianglai Biological Technology Co; cat. no. AF0166). The presence of yellow or brownish-yellow particles indicated immunopositivity. Positive cells in 5 high-power random fields in each tissue section were counted. Positive expression was graded based on the percentage of positive cells and staining intensity as follows: negative (−), <10% positive cells and light yellow color; weak positive (+), 10%–25% positive cells and yellow to brown color; strong positive (+++), >50% positive cells and brownish yellow to tan color; and positive (++), intermediate between (+) and (+++).

### 16S rRNA gene sequencing

Intestinal mucosa tissue samples that were not used for routine pathologic examination were immediately frozen in liquid nitrogen and stored at −80°C until analysis. The whole blood sample remaining after clinical testing was centrifuged, and the serum was stored at −80°C. Genomic DNA extraction and PCR amplification were performed by Jiangsu Huada (BGI) Chemical Group Co, Changzhou, China, and Meiji Biotechnology, Shanghai, China). The purity of the extracted DNA was confirmed by 1% agarose gel electrophoresis. PCR amplification was performed using TransStart FastPfu DNA polymerase (TransGen Biotech, Beijing, China; cat. no. AP221-022) on a GeneAmp 9700 thermal cycler (Applied Biosystems, Foster City, CA, USA). Quantitative fluorescence electrophoresis was performed for preliminary quantitation of the PCR products using the QuantiFluor-ST system (Promega, Madison, WI, USA). After library construction, the V3–V4 hypervariable region of the bacterial 16S rRNA gene was amplified using the following primer pair: 338-ACTCCTACGGGAGGCAGCAG (forward) and 806-GGACTACHVGGGTWTCTAAT (reverse). Because of a conflict in sample delivery time, 16S rRNA gene sequencing was performed by Majorbio Bio-Pharm Technology Co. (Shanghai, China) and Huada (BGI) Biotechnology Co (Wuhan, China). The purified amplifiers were mixed at an equimolar ratio and sequenced with the MiSeq platform (Illumina, San Diego, CA, USA) in the paired end (PE) 300 mode.

### Bioinformatics analysis

PE reads obtained by library sequencing were stitched together based on overlapping regions. Sequence quality was controlled and filtered, and OTU clustering and taxonomic analysis were performed. We extracted nonrepeating sequences as optimized sequences to reduce redundancy calculations in the analysis, removed single sequences without repetitions, and performed OTU clustering on nonrepeating sequences (excluding single sequences) based on 97% similarity. Chimeras were removed in the clustering process, yielding representative OTU sequences. We mapped all optimized sequences to representative OTU sequences and selected those with >97% similarity to representative sequences to generate OTU tables.

We experimentally grouped factors in the original data that were quality controlled and carried out species annotation and composition analysis (including OTU and α diversity analyses). We then constructed a heatmap of the community structure and Circos diagram of the relationship between samples and species and carried out comparative analyses of the sample, including PCoA and multigroup, intergroup, and LEfSe multilevel analyses of species differences. Finally, we performed a correlation analysis of clinical data, constructed a heatmap of the correlation, and carried out linear regression analysis on 5 indices with significant correlations.

### Statistical analysis

Statistical analyses were performed using R software package (R Core Team, 2013) and SPSS v19.0 software (SPSS Inc, Chicago, IL, USA). Differences in Sobs, Shannon, and ACE indices between groups were evaluated for statistical significance with the Wilcoxon rank-sum test, and LEFSe was used to identify species responsible for the differences, with a nonparametric factorial Wilcoxon rank-sum test used to assess differences in abundance. Species differences between groups were evaluated with the Kruskal–Wallis test. Differences in serum GLP-2 expression were analyzed with the independent samples t-test. The F statistic for the linear regression model was based on sample variance analysis.

## Results

The previous studies shown that GLP-2 is synthesized in the enteroendocrine L cells (along with GLP-1) and in the brainstem and hypothalamus of the central nervous system. GLP-1 mainly regulate insulin secretion in beta cells of pancreatic islets, where GLP-2 widely regulate intestinal physiological functions.

### Characteristics of the study population

The characteristics of the study population are shown in Table 1. There were no significant differences in age or body mass index (BMI) between UC patients and healthy control subjects.

### Intestinal microbiota diversity and abundance are reduced in UC

**Figure 1.**
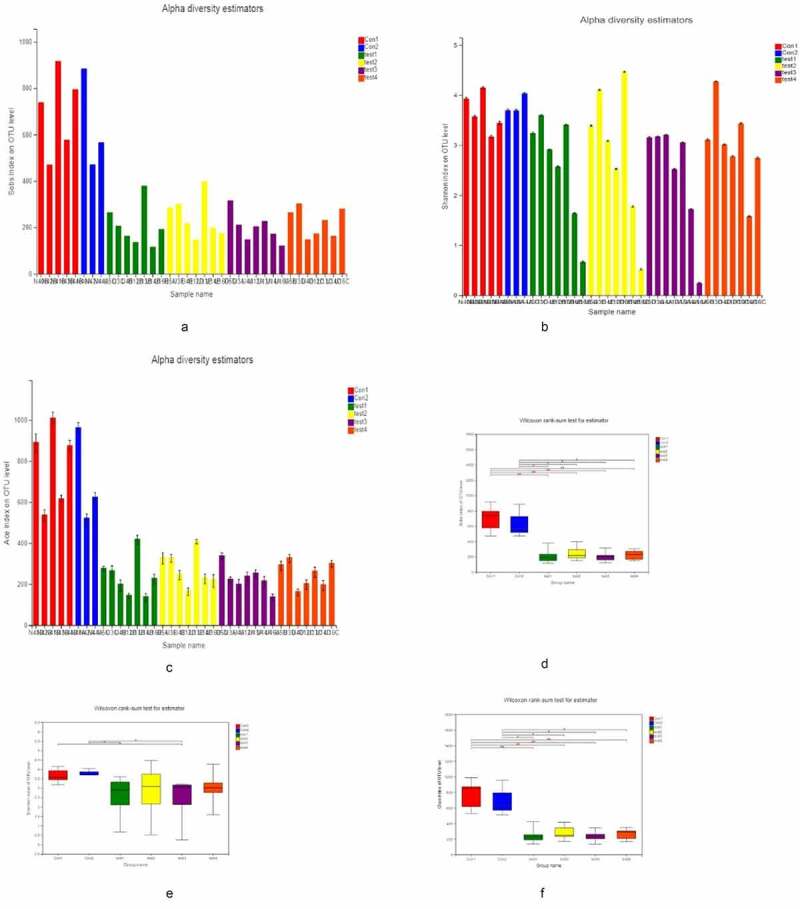
**α Diversity analysis** A, B, C for **So**bs, Shannon, and ACE indices of UC patients and healthy control subjects at the OTU level. D, E, F for Significant differences between samples from 2 selected groups. *P < 0.05, **P < 0.01, ***P < 0.001. X and Y axes show group names and exponential averages for each group, respectively

We divided the tissue samples into the following 6 groups for 16S rRNA gene sequencing and bioinformatics analysis (Figure 1): normal ileum; normal colon; normal tissue from UC patients; lesions from UC patients; UC patient ileum; and UC patient junctional area. We assessed the α diversity of the samples based on observed subsample species richness (Sobs), which was used to estimate the richness of operational taxonomic units (OTUs); the Shannon index and abundance-based coverage estimator (ACE) index were used to measuring species diversity and richness, respectively (Figure 1a–C). The Sobs, Shannon, and ACE indices were significantly lower in UC patients than in healthy subjects, as determined with the Wilcoxon rank-sum test (Figure 1d–F). However, there were no significant differences in these indices among UC patient groups.

**Figure 2.**
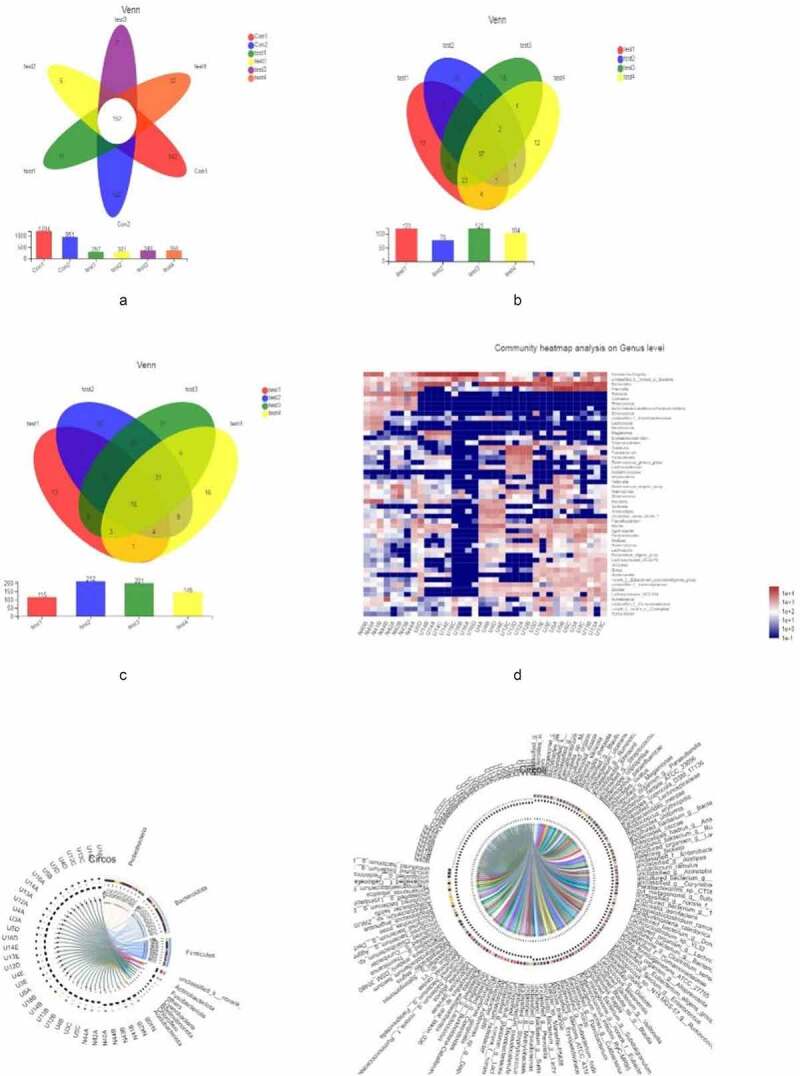
**Microbiota composition,A, B, C** represent the Venn diagram with different colors representing different groups (or samples); numbers in overlapping areas represent the number of species common to multiple groups, and numbers in nonoverlapping areas represent the number of unique species in corresponding groups. **D**, the X and Y axes show sample or group names and species names, respectively. The abundances of different species in the samples are represented by a color gradient; values represented by the color are shown to the right. **E, F** represent the Circos diagram of the relationship between sample and species

We then examined overall microbiota composition in the 6 groups by quantifying the number of different taxa. In order to eliminate the influence of environmental factors, we also compared samples showing significant differences in composition within each group. Compared to the normal control group, the abundance of mucosal microbiota was reduced in UC patients, although there were no differences in microbiota diversity or abundance among UC patients (Figure 2a–c). The similarities and differences in gut microbiota composition were clearly observed in the heatmap (Figure 2d); Proteobacteria, Bacteroides, Firmicutes, Unclassified_k_norank_d_Bacteria, Actinobacteria, and Fusobacteria showed differences between UC patients and healthy controls (Figure 2e, f).

We carried out a β diversity analysis to evaluate differences in gut microbiota composition between samples (Figure 3). The principal coordinate analysis (PCoA) showed that at the phylum level, there were significant differences in bacterial composition between UC and normal control groups (Figure 3a, b). However, there were no differences among UC patients. In order to evaluate the relationship between species composition and lesion severity, we used linear discriminant analysis effect size (LEFSe) and the nonparametric Kruskal–Wallis test to identify species responsible for intergroup differences (Figure 3c). Compared to normal control samples, UC patients showed reduced microbiota diversity and abundance in the colon irrespective of lesion severity, with significant differences observed at the phylum level (Actinobacteriota, Chloroflex, and Patescibacteria) and genus level (*Ralstonia* and *Rhodococcus*, which were less abundant in UC patients) (Figure 4a, b). Lower diversity and abundance of microbiota was also observed in the small intestine of UC patients, with significant differences for Acidobacteria, Planctomycetota, and other phyla (Figure 4c, d). Among UC samples, there were differences between relatively normal tissue and lesions at the phylum (e.g., Actinobacteriota and Chloroflexi) and genus (eg, Ralstonia, Rhodococcus, Burkholderia-Caballeronia-Paraburkholderia) levels (Figure 4e, f). Thus, differences in intestinal mucosa microbiome profiles at the phylum and genus levels in UC patients were independent of lesion severity (Figure 4g, h).

**Figure 3.**
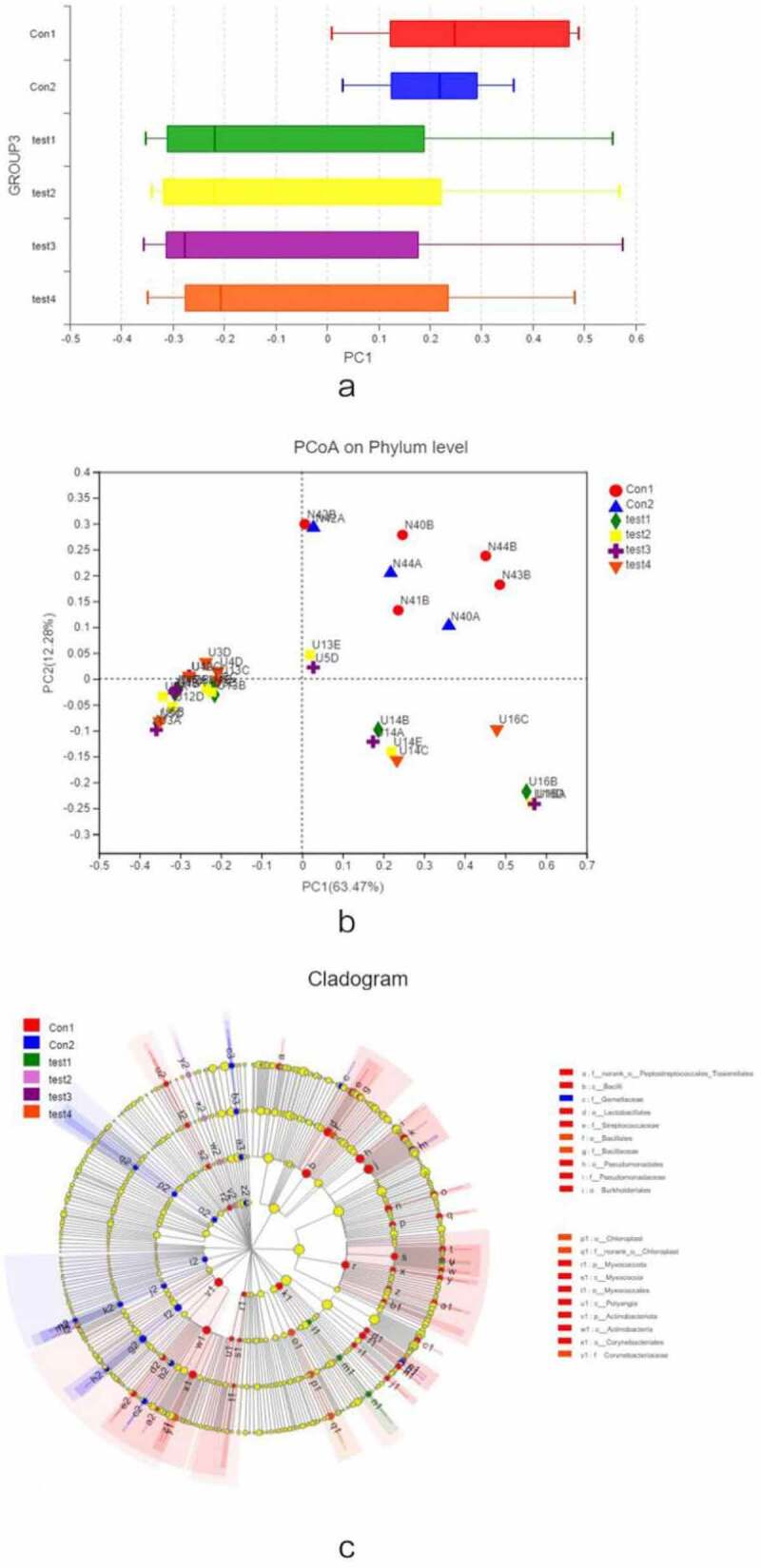
**β diversity analysis, A**, different colors represent sample groupings, and the box chart in the figure represents the dispersion of different groups of samples on the PC1 axis. **B**, X and Y axes show 2 selected spindles, and the percentages are the interpreted value of the axes for differences in sample composition. The scales of the X and Y axes are relative distances that have no practical significance. Different colors or shapes represent different groupings of samples; a shorter distance between 2 sample points indicates greater similarity in their species composition. **C**, different color nodes represent microbiota that is significantly enriched in the corresponding group and have a significant effect on intergroup differences; pale yellow nodes represent microbiota that are not significantly different across groups or that have no significant effect on intergroup differences

**Figure 3.**
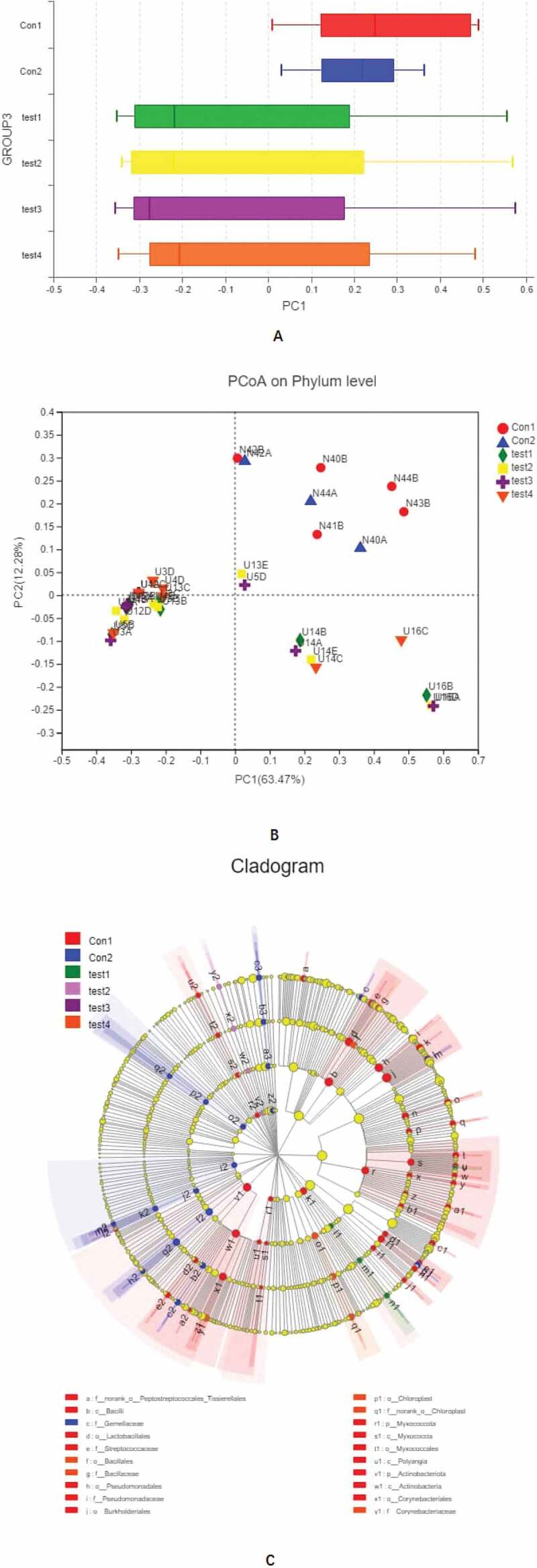
Continued

**Figure 4.**
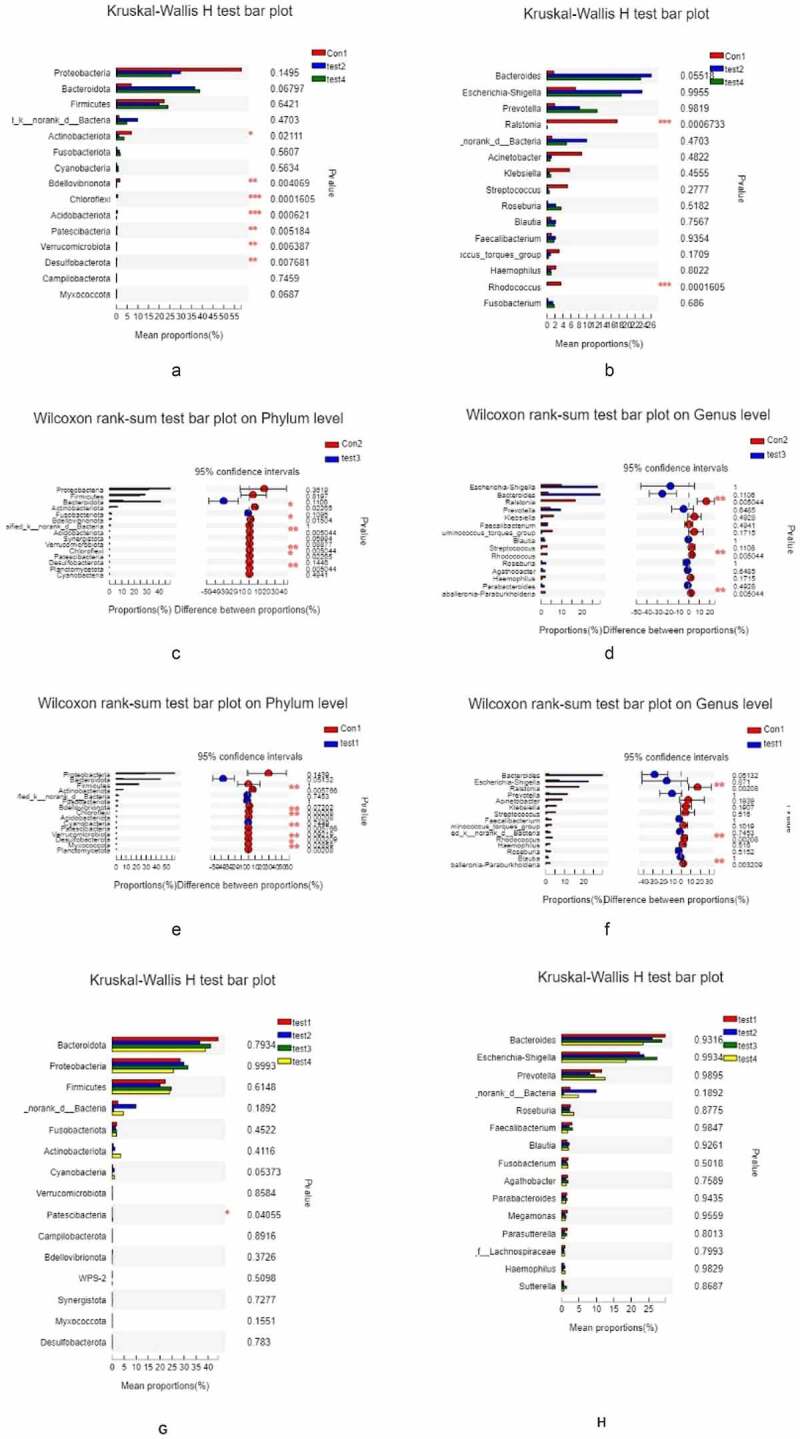
**Relationship between species composition and lesion severity**. Compared to normal control samples, UC patients showed reduced microbiota diversity and abundance in the colon irrespective of lesion severity, with significant differences observed at the phylum level (Actinobacteriota, Chloroflex, and Patescibacteria) and genus level (Ralstonia and Rhodococcus, which were less abundant in UC patients) (a, b). Lowerdiversity and abundance of microbiota was also observed in the small intestine of UC patients, with significant differences for Acidobacteria, Planctomycetota, and other phyla (c, d). Among UC samples, there were differences between relatively normal tissue and lesions at the phylum (e.g., Actinobacteriota, and Chloroflexi) and genus (eg, Ralstonia, Rhodococcus, Burkholderia-Caballeronia-Paraburkholderia) levels (e, f). Thus, differences in intestinal mucosa microbiome profiles at the phylum and genus levels in UC patients were independent of lesion severity (g, h)

### Glucose metabolism and biochemical indices are perturbed in UC

**Figure 5.**
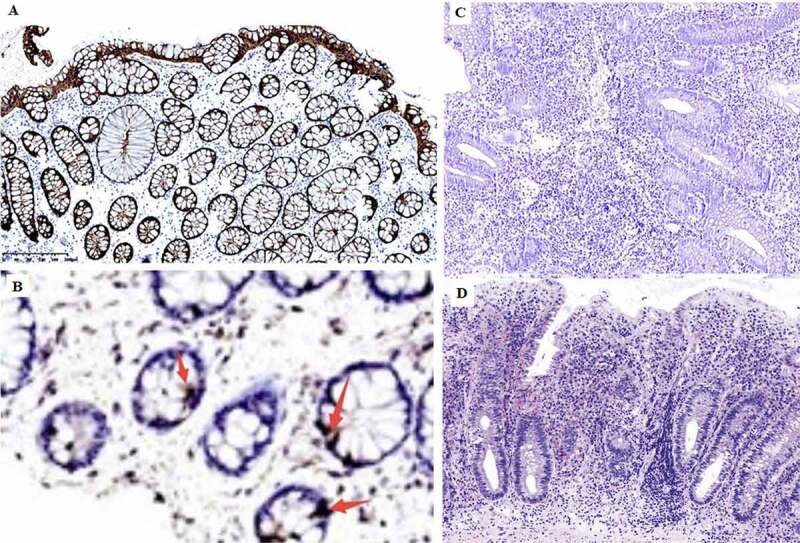
Immunohistochemical detection of GLP-2 expression in the intestinal mucosa. **A**, strong positive expression of GLP-2 (+++) (most healthy control samples). B, weak positive expression of GLP-2 (+). C, negative expression of LP-2 (−) (most UC samples), D, diagnosed UC sample histology H&E staining

In order to determine whether glucose metabolism in the gut is altered in UC, we measured serum levels of GLP-2 by enzyme-linked immunosorbent assay (ELISA). GLP-2 level was significantly lower in UC patients than in healthy control subjects (P < 0.01; Tables 2, 3). The lower level of serum GLP-2 in UC was correlated with the observed reductions in microbiota diversity and abundance. To confirm these results, we performed an immunohistochemical analysis of GLP-2 expression in mucosal tissue samples and found that GLP-2 was expressed in the glands of the intestinal epithelium and was localized in the cell membrane and cytoplasm; however, the expression was reduced in UC patients compared to healthy control subjects (P < 0.05; Figure 5, Table 4).

**Table 2.** Optical density values for serum GLP-2 detected by ELISA in ulcerative colitis patients and healthy control subjects

**Group^†^**	**N**	**Mean**^‡^	**Standard deviation**	**Standard error of the mean**
1	11	0.32223636	0.100551340	0.030317370
2	9	0.51012778	0.112026674	0.037342225

^†^Group 1, ulcerative colitis patients; Group 2, healthy control subjects.

^‡^Values for both groups conformed to a normal distribution, and statistical significance was evaluated with the independent samples t-test(p < 0.05) between groups 1 and 2.

ELISA, enzyme-linked immunosorbent assay; GLP-2, glucagon-like peptide 2.

**Table 3.** Results of the independent samples test for optical density value of serum GLP-2 in ulcerative colitis patients and healthy control subjects

	Levene’s test of variance		t-test of mean					
	**F**	**Significance**	**t**	**df**	**P-value (2-sided)**	**Mean difference**	**Standard error**	**95% CI of mean difference**
Equal variance assumed	0.501	0.488	−30.951	18	0.001	−0.187891414	0.047555924	−0.087980124, −0.287802704
Unequal variance assumed			−30.906	160.342	0.001	−0.187891414	0.048099737	−0.086097686, −0.289685143

CI, confidence interval; GLP-2, glucagon-like peptide 2.

**Table 4.** The expression of Glp-2 in colonic mucosa group number (-) (+) (++) (+++) UC 11 4 3 3 1 Normal 9 0 2 4 3

Chi-squared Test					
	value	df	ProgressiveSig. (bilateral)	Precision Sig.(bilateral)	Precise Sig.(unilateral)
Pearson chi-square	16.000a	1	.000		
continuous	12.195	1	.000		
Correction Likelihood ratio	21.930	1	.000		
Fisher’s exact test				.000	.000
Linear and linear combination	15.000	1	.000		
N in a valid case	16				

Note: Because the expected count was less than 5, the sample size was insufficient, but Fisher’s exact probability method could be used to test, and the positive rate difference between the two groups was statistically significant (P = 0.00).

### Alterations in glucose metabolism and inflammation are associated with microbiome profile in UC

**Figure 6.**
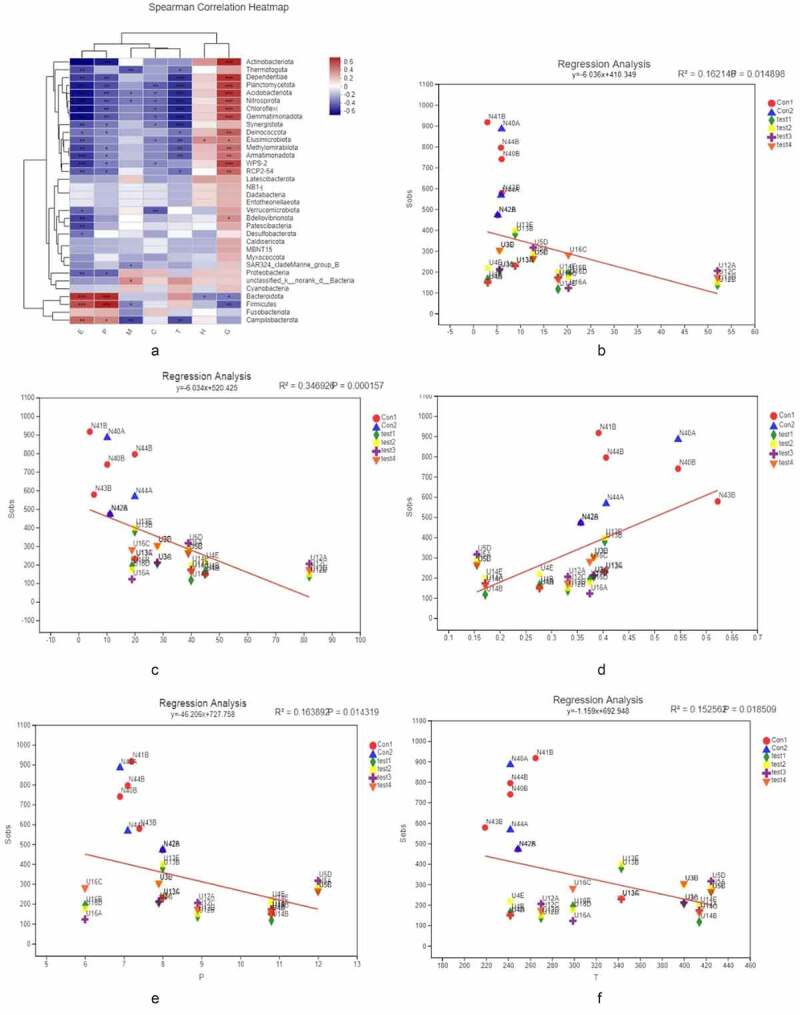
**[α diversity]**. a X and Y axes show clinical parameters and bacterial species present in the intestinal mucosa, respectively. *P < 0.05, **P < 0.01, ***P < 0.001. b–f X and Y axes show clinical parameters and β – or α-diversity index, respectively. The X-coordinates represent environmental factors (e.g., pH, temperature, etc.), the Y-coordinates represent the rank of β-diversity or the α-diversity index, R2 is a determining factor, representing the proportion of variation interpreted by the linear regression line, and the greater R2 index, the higher the degree of interpretation of this environmental factor is on the differences in the composition of the group or in the α-diversity index. b C-reactive protein. c Erythrocyte sedimentation rate. d Thrombocyte count. e GLP-2. f OGTT

To assess the severity of inflammation in the intestinal mucosa of UC patients and its impact on gut microbiome profile, we examined indices of intestinal epithelium inflammation in UC, including erythrocyte sedimentation rate, C-reactive protein, hemoglobin (Hb), thrombocyte count, neutrophil granulocyte percentage, and the optical density (OD) value of GLP-2 in ELISA. Glucose tolerance was evaluated with the oral glucose tolerance test (OGTT), and a correlation heatmap was generated to visualize the relationship between microbiota species and the aforementioned markers (Fig. 6A). Hb and neutrophil granulocyte percentage, as well as other physiologic parameters, were related to changes in the abundance of Actinobacteria, Dependentiae, Planctomycetota, Gemmatimonadota, etc. To further validate this result, we carried out a linear regression analysis of 5 clinically relevant parameters (erythrocyte sedimentation rate, C-reactive protein, thrombocyte count, and the optical density (OD) value of GLP-2 in ELISA. Glucose tolerance was evaluated with the oral glucose tolerance test (OGTT). We first performed PCoA (全名), in which the score (β-diversity or the α-diversity index) of each sample and the parameters (e.g, OD of GLP-2, platelet count, etc.) were plotted on the Y and X axes of the scatter plot, respectively; we then performed linear regression to evaluate the relationship between the parameters. We found that alterations in gut microbiome profile were positively correlated with GLP-2 level and negatively correlated with the other clinical indices, and the correlations were strengthened with greater disease severity (Fig. 6B–F).

## Discussion

In this study, we analyzed gut microbiome profiles in different parts of the intestinal mucosa with lesions of varying severity and examined whether these are related to clinical features of UC. To our knowledge, this is the first study in which biopsies obtained from different parts of the intestine were compared. Based on high throughput 16S RNA sequencing and glucose metabolism analyses, we first examined microbiota from intestinal areas with severe lesions, borderline lesions, and relatively normalfrom patients with segmental distribution of UC; these were also compared to the corresponding area in biopsy samples from healthy controls. The results showed that both the diversity and overall abundance of microbiota in the ileum and colon were significantly reduced in UC patients compared to healthy subjects. Similar trend was observed in earlier work [19], but our results contradict findings from a previous study that showed by quantitative PCR analysis that severe UC was associated with enrichment of bacteria on the mucosal surface [20] Increased mucosal secretion and necrosis can lead to enrichment of gut microbiota, which can colonize host tissues and induce an immune response [21].

Contrary to our expectation, there were no differences in microbiota diversity and abundance between samples from different parts of the gut or according to lesion severity in individual patients or among patients with UC, which is in accordance with a previous report [22]. These results suggest that the gut microbiome profile is not the main factor influencing the degree of local inflammation, although this requires confirmation in future studies.

We compared microbiota at the terminal ileum of UC patients and healthy control subjects. The ileum in UC patients was normal (no ulcerative lesions) and lesions were ‘inverted’ and far away from the cecum; nonetheless, the diversity and abundance of microbiota at the terminal ileum were significantly reduced regardless of whether a lesion was present, as reported by others [23,24]. Colonization by microbiota is influenced by the tissue microenvironment, mainly by factors such as oxygen gradient, protein content, etc. Disruption of the mucosal barrier and loss of bacterial diversity at the terminal ileum are potential indicators of lesions along the entire colon [25].

We performed intergroup and intragroup comparisons of intestinal mucosa samples by (全名) analysis from the phylum to species level. Although our results markedly diverged from those obtained using fecal samples, they were consistent with other reports [26,27]. There were no differences in microbiota diversity or abundance according to lesion severity in UC, and the species found in UC samples were the same as those present in healthy control subjects as previously demonstrated [28]. Even the invasive and adhesive enteropathogenic bacterium *Escherichia coli* was not detected in lesions from UC patients, although it is present in both the small and large intestines of patients with Crohn’s disease [29,30]. This implies that gut bacteria do not promote the occurrence of UC through local colonization and induction of the host immune response but instead cause inflammation by altering metabolite levels and weakening the mucosal barrier [25].

We examined serum GLP-2 levels and found that it was correlated to reduced microbiota diversity and abundance in UC. Abnormal glucose metabolism has been linked to UC pathogenesis, and GLP-2 is downregulated in patients with UC. GLP-2 is a secreted protein whose level was found to be positively correlated with the abundance of gut microbiota, with a stronger correlation observed in more severe disease. The blood glucose level in the OGTT was the main factor affecting lesion severity in UCand was related to the reduced diversity of microbiota. Thus, decreases in the diversity and abundance of gut microbiota may result in decreased levels of protective factors and negative effects on host metabolism. ErbB (全名) signaling was shown to regulate the intestinal secretion of GLP-2 [14,31]; and the Janus kinase (JAK)/signal transducer and activator of transcription (STAT) signaling pathway – the main pathway dysregulated in UC – influences the activation of epidermal growth factor (EGF)/ErbB receptor [32]. Microbiota can directly modulate JAK/STAT signal transduction [33] thus, the relationship between the gut microbiome, glucose metabolism, and UC can be summarized as follows: alterations in microbiota diversity and abundance induce the activation of redox, JAK/STAT, and EGF/ErbB signaling, upregulation of GLP-2, and pathogenic changes in the intestinal mucosa.

Although this study was limited by small sample size, it nonetheless demonstrates that colonization by specific microbiota is not the main determinant of lesion severity in UC. Moreover, a reduced abundance of certain bacterial taxa at the terminal ileum can be an early indicator of the disease. GLP-2 is closely related to the metabolic activity of bacteria and has a protective effect against inflammation. Thus, therapeutic strategies that increase GLP-2 levels may be effective in the treatment of UC.

## Conclusions

GLP-2, a peptide hormone secreted by L-cells in intestinal mucosa may be effective in the treatment of UC due to its increasing roles in intestinal microbiota, and hormone functions which can be easily expanded.

## Data accessibility

Raw sequences generated by 16S rRNA sequencing have been deposited in the NCBI Sequence Read Archive (SRA: SUB8157907; Study PRJNA664267), and the relevant metadata can be found at https://www.ncbi.nlm.nih.gov/Traces/study/ under the SRA accession number.
